# Anti-Obesity and Lipid Metabolism Effects of *Ulmus davidiana* var. *japonica* in Mice Fed a High-Fat Diet

**DOI:** 10.4014/jmb.2102.02015

**Published:** 2021-05-28

**Authors:** Sung-Gyu Lee, Hyun Kang

**Affiliations:** Department of Medical Laboratory Science, College of Health Science, Dankook University, Cheonan 31116, Republic of Korea

**Keywords:** *Ulmus davidiana* var. *japonica*, (+)-catechin, anti-obesity, 3T3-L1, high-fat diet

## Abstract

The root bark of *Ulmus davidiana* var. *japonica* (Japanese elm) is used in Korea and other East Asian countries as a traditional herbal remedy to treat a variety of inflammatory diseases and ailments such as edema, gastric cancer and mastitis. For this study, we investigated the lipid metabolism and anti-obesity efficacy of ethyl alcohol extract of *Ulmus davidiana* var. *japonica* root bark (UDE). First, HPLC was performed to quantify the level of (+)-catechin, the active ingredient of UDE. In the following experiments, cultured 3T3-L1 pre-adipocytes and high-fat diet (HFD)-fed murine model were studied for anti-obesity efficacy by testing the lipid metabolism effects of UDE and (+)-catechin. In the test using 3T3-L1 pre-adipocytes, treatment with UDE inhibited adipocyte differentiation and significantly reduced the production of adipogenic genes and transcription factors PPARγ, C/EBPα and SREBP-1c. HFD-fed, obese mice were administered with UDE (200 mg/kg per day) and (+)-catechin (30 mg/kg per day) by oral gavage for 4 weeks. Weight gain, epididymal and abdominal adipose tissue mass were significantly reduced, and a change in adipocyte size was observed in the UDE and (+)-catechin treatment groups compared to the untreated control group (***p < 0.001). Significantly lower total cholesterol and triglyceride levels were detected in UDE-treated HFD mice compared to the control, revealing the efficacy of UDE. In addition, it was found that lipid accumulation in hepatocytes was also significantly reduced after administration of UDE. These results suggest that UDE has significant anti-obesity and lipid metabolism effects through inhibition of adipocyte differentiation and adipogenesis.

## Introduction

Herbal medicines, which can be defined as functional raw or extracted materials isolated from plants, have lower side effects than pharmacological drugs, so they are widely used in the prevention and treatment of chronic diseases such as obesity [1]. *Ulmus davidiana* var. *japonica*, a widely used herb in oriental medicine, and its root bark, known as yugeunpi. It contains anti-inflammatory anti-microbial and anti-cancer properties [2-6]. It has also been used in herbal medicines for the use of edema, stomach cancer, mastitis and inflammation [4]. Several flavonoids [7] and terpenoids [8] identified in the stem and the root barks of *U. davidiana* var. *japonica* has been evaluated for pharmacological action. However, most studies have reported on anti-oxidant effects in vitro, and in vivo studies are very limited. Although hyperlipidemia is potentially associated with obesity, there are few studies on the anti-obesity effects of *U. davidiana* var. *japonica*. The main characteristics of obesity that cause all diseases are excessive weight and abnormal body fat accumulation [9]. Obesity, in particular, has been shown to be closely related to the induction of several metabolic diseases, such as hyperlipidemia, insulin resistance, and high blood pressure [10]. Obesity is a global problem, and its prevalence is increasing, making it a social problem. Currently, both Orlistat and Sibutramine, are approved for long-standing cure of obesity. However, these drugs have side effects along with elevated blood pressure, oral dehydration, constipation, insomnia and headache [11]. Researching potential natural products for anti-obesity efficacy is currently being explored. It could be an alternative strategy for improvement of safer and more effective anti-obesity drugs [12].

Adipogenesis is the development of preadipocytes into mature adipocytes, a process particularly involved in controlling lipid droplet size (hypertrophy) as the number or proliferation of cells increases. Synthesis of adipocytes plays a key role in obesity. Therefore, the adipogenic process can be studied using pre-adipocyte cell lines as valuable models. This process can provide a benefit by preventing the beginning and development of adipogenesis, which is a clue in human obesity and obesity-related diseases [13]. Different transcription factors, including peroxisome proliferator-activated receptor γ (PPARγ), which is regarded as the "master regulator of fat frormation", are involved in the initiation of differentiation of pre-adipocytes and influence adipogenesis [14].

The transcription factor for adipogenesis involves and regulates the CCAAT / enhancer binding protein (C /EBP) family and the sterol regulatory element binding protein 1c (SREBP-1c). In this study, a 3T3-L1 cell model and a high-fat diet (HFD) induced obesity rat model were tested to determine the anti-obesity effect. We demonstrated that UDE has a significant anti-obesity effect by regulating lipid metabolism in both differentiated adipocytes and HFD-induced obese mice.

## Materials and Methods

### Preparation of UDE

*U. davidiana* var. *japonica* was purchased at the herbal medicine market in Seoul, South Korea. 1 kg was extracted with 10 L of 70% ethanol for 24 hours at room temperature (RT). The ethanol extraction supernatant was filtered through filter paper (Whatman No. 1 & 3; Whatman, UK) and separated using a rotary vacuum vaporizer (N-1000S-WD; Eyela Co., Japan). The yield (w/w) obtained with UDE was 12.4%.

### High-Performance Liquid Chromatography (HPLC)

To quantify the level of (+)-catechin (Sigma-Aldrich Co., USA) as a marker component of UDE, a Waters 2690 (USA) HPLC system equipped with an autosampler was used with a 20 μl loop. Chromatographic analysis was performed using a Gemini C_18_ reverse-phase column (4.6 mm × 100 mm) filled with 3 μm diameter particles. The two mobile phases used were as follows: solvent A, 0.9% acetic acid in H_2_O; solvent B, 100% acetonitrile. Gradient flow was used from 95:5 (solvent A: solvent B vol%) to 65:35 (solvent A: solvent B vol%) of at a gradient time of 35 min at a run rate of 1.0 ml/min. The column temperature was set to 33°C. The wavelength used for detection was of 280 nm. The identification of (+)-catechin isolated from UDE is based on the retention time compared to the standard. The concentration of (+)-catechin was confirmed by a standard curve and expressed as mg/100 g of dry weight.

### Cell Culture

3T3-L1 pre-adipocytes were gotten from American Type Culture Collection (ATCC, USA) and cultivated. Cells were cultured and used in a CO_2_ cell culture incubator at 37°C using Dulbecco's modified Eagle's medium (DMEM) and 10% calf serum (BCS), 100 U/ml penicillin, and 100 μg/ml streptomycin. After cells about 80%confluence, adipocyte differentiation was induced by treatment of 3-Isobutyl-1-methylxanthine (IBMX , 23 mg/ml), dexamethasone (1 mM), and insulin (1 mg/ml), in DMEM plus 10% BCS. After forty-eight hours, the stimulation medium was changed with DMEM plus insulin (10 mg/ml). New supplemented medium was added after two days, at which phase more than 90% of cells were matured adipocytes involve accumulated fat droplets. To determine the effect of UDE and (+)-catechin on adipocyte differentiation, the 3T3-L1 cells were treated with 10, 25, 75, 100, or 200 μg/ml UDE and 50 μM (+)-catechin every two days starting two days post-confluence up to the end of the examination on day six.

### Cell Viability

Cell viability was evaluated using (3-(4,5-Dimethylthiazol-2-yl)-2,5-Diphenyltetrazolium Bromide (MTT) analyze. Cells (1 × 10^6^ cell/well) were added in a 96-well dish and treated with UDE and (+)-catechin. After 48 h, MTT solution (5 mg/ml) made with 10 μl of phosphate buffered saline (PBS) was treated into individual wells of the plate and incubated at 37°C for 4 h. Then, 100 μl of dimethyl sulfoxide (DMSO) was dissolved in MTT formazan and then analyzed at an absorbance of 550 nm using a microplate reader (Bio-Rad Laboratories Inc., USA).

### Oil Red O Staining

Cell lipid accumulation in adipocytes was determined by Solvent Red 27, Sudan Red 5B, C.I. 26125, C26H24N4O (Oil Red O). Cells were added as a control or adipogenesis from days 0 to 2 with 10, 25, 75, 100 or 200 μg/ml UDE and 50 μM (+)-catechin. The medium was changed every 2 days. On day 6 of differentiation, cells were washed with phosphate buffered saline (PBS), treated with 10% formaldehyde for 1 hour, and then washed with 60% isopropanol. Cells were stained for 1 hour using a 0.3% Oil Red O staining solution (7 parts of water in isopropanol and 3 parts of 0.3% Oil Red O dye), washed 3 times with water, and then photographed under a microscope. Oil Red O was also dissolved using 100% isopropanol and measured at 520 nm.

### Western Blot Analysis

Differentiated 3T3-L1 adipocytes were treated with different concentrations of UDE and (+)-catechin. After 48 hours, cells were harvested and washed with PBS. Cells and liver tissue were lysed in RIPA buffer (including phosphatase and protease inhibitor). 3T3-L1 cells were extracted for 30 min at 4°C. Then, centrifugation was performed at 18,928 ×*g* for 15 min to obtain a supernatant. Protein concentration in the supernatant was determined using a protein assay kit. 10 μg of cellular protein was electrophoresed by 10% sodium dodecyl sulfate-polyacrylamide gel electrophoresis (SDS-PAGE) gel electrophoresis and transferred to Immobilon-P-membrane (Millipore, USA). Western blot membranes reacted with specific antibodies (PPAR-γ, SREBP-1c and C/EBP-α), allowing for chemiluminescence (ECL).

### Real-Time Polymerase Chain Reaction (RT-PCR)

Total RNA was extracted from differentiated 3T3-L1 adipocytes using TRIzol reagent (ThermoFisher Scientific, USA). The reverse transcription test of total RNA was performed using AccuPower RT PreMix (Bioneer, Inc., USA). CDNA was obtained by reverse transcription of the total RNA of cells using 2 μg of total RNA in a final reaction volume of 20 μl using the High Capability RNA-to-cDNA Kit (Bioneer, Inc., Korea) according to the manufacturer's manufacturing instructions. Reverse transcription were carried out with incubation for 1 h at 42oC, followed incubation for 5 min at 99oC. RT-PCR amplification was performed using 3 μl cDNA diluted 1:20 with each target gene specific primer group. Table 1 shows the primer sequences of sense and antisense oligonucleotides recycled for amplification. Each set of oligonucleotides was recycled to a concentration of 100 μM in a final volume of 14 μl using SYBR Premix Ex Taq DNA Polymerase (Takara Biotechnology Co., Ltd., Japan). PCR reactions were performed using a fluorometric thermal cycler (Thermal Cycler Dice Real Time System); Takara Biotechnology Co., Ltd.). The Tbp hose keepin gene was used for normalization of Ct values. Oligonucleotide sequences are presented in Table 1.

### Animals and Experimental Design

C57BL/6J mice (age five-six weeks, weight 20-22 g) were purchased from DBL Inc. (Korea). All study animals were housed in a group (*n* = 6) under 12 hour light and dark cycle at room 23 ± 2°C and 50 ± 10% humidity. They were fed a 10 kcal% low fat diet (LFD; Research Diet, New Brunswick, NJ, USA) or 60 kcal% HFD (Research Diet) during the 8-week experimental period. The animals were subdivided into four groups and fed for 4 weeks before being sacrificed: LFD group, HFD group, HFD plus 200 mg/kg/day UDE (HFD-UDE group), and HFD plus 30 mg/kg/day (+)-catechin (HFD-C group). Before victim, their feed was withdrawn for twelve hour. After all mice used in the experiment were euthanized using isoflurane, cells and tissues were separated, weighed, flash frozen in liquid nitrogen and stored at -80°C until testing. Body weight and dietary intake were recorded weekly and every two days. The anti-obesity murine model study was approved by the animal experiment committee of Dankook University (DKU). Study animals were performed according to the guidelines of DKU's Animal Experiments Committee (DKU-18-019).

### Body Weight Gain and Feed Efficiency Ratio

The weight of the mice in the experiment was measured daily by blind individuals in each experimental group. Feed intake was measured by approximating the amount of feed consumed by mice during the experiment. Food spillage was investigated in the experimental cage, but only a small amount of spillage was observed, and feed intake was also harvested. The feed efficiency ratio (FER) was obtained as follows:

FER (%) = body weight gain (g/d) / feed intake (g/d) × 100

After fasting for 12 hours on the last day of the experiment period, the mice were sacrificed for cervical destruction.

### Tissue Preparation and Analysis of Plasma Lipid Profiles

At the end of the 4-week UDE dosing period, mice were sacrificed under isoflurane anesthesia after an overnight fast, and blood was collected in heparin tubes. The blood collected from the mice was centrifuged at 1,500 ×*g* for 15 min, and the plasma was stored at -70 °C until the test. The epididymis and abdominal fat of the test mice were immediately separated, washed with ice-cold saline, and weighed respectively. The concentrations of liver biomarkers, plasma alanine transaminase (ALT), aspartate aminotransferase (AST), and lipid biomarkers, total cholesterol (TC) and triglycerides (TG), were measured with a Konelab20XT automated blood analyzer (Thermo).

### Histopathological Analysis

After the mice are sacrificed, the abdominal tissue is separated and made into small pieces. After fixing for 24 h in a 10% formaldehyde solution neutralized to pH 7.4, the tissue block is embedded in a paraffin block and successively cut into 4 μm thick slices. Place in a plain glass, deparaffinize twice for 5 min in xylene, then rehydrate with a graded alcohol series. Slides containing sliced tissue were stained with Mayer's hematoxylin and eosin (H&E) and measured at 400× magnification using an optical microscope.

### Statistical Analysis

The experimental results used in this study were expressed as mean ± standard deviation (SD) and analysis of variance was used for statistics. Duncan's multi-range test was performed to define the significant variance between groups. In the results of this study, *p* <0.05 was considered significant.

## Results

### (+)-Catechin Content in UDE

**Fig. 1:**
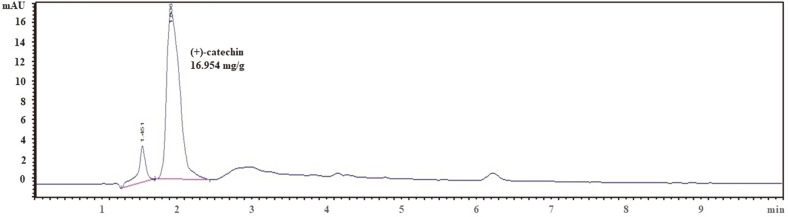
Identification of (+)-catechin in UDE using HPLC chromatogram.

The analytical HPLC chromatogram of (+)-catechin in the UDE is shown in Fig. 1. A calibration curve was established for the (+)-catechin standard. The (+)-catechin content of the UDE was 16.954 mg/g (data not shown).

### Cell Viability and Lipid Accumulation by UDE and (+)-Catechin in 3T3-L1 Pre-Adipocytes

**Fig. 2:**
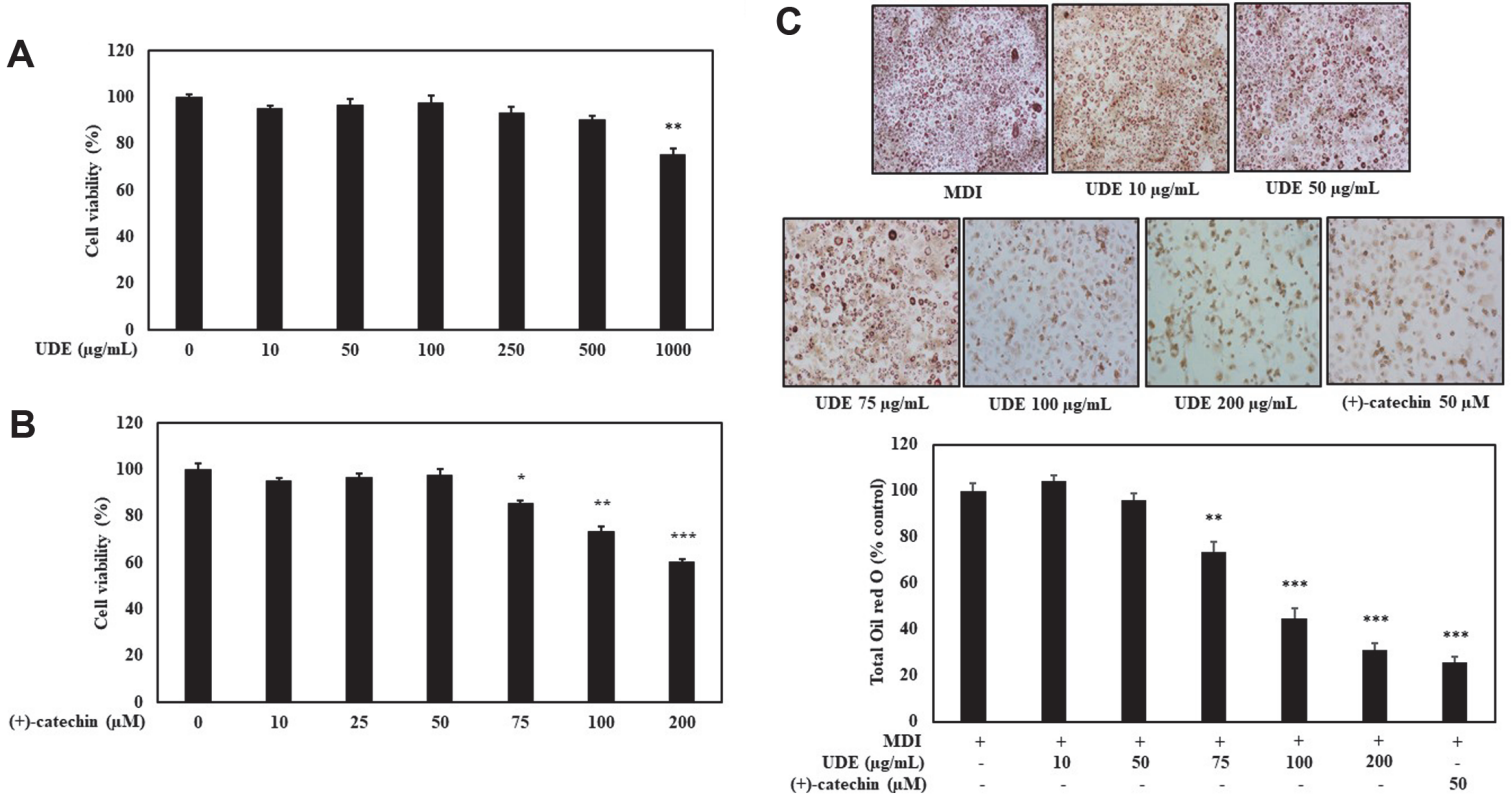
Effects of UDE and (+)-catechin on cell viability and differentiation of 3T3-L1 preadipocytes. (**A**) An MTT assay was performed for analysis of cell viability after treatment with UDE (10, 50, 100, 250, 500, and 1,000 μg/ml) or (**B**) (+)-catechin (10, 25, 50, 75, 100, and 200 μM) for 48 h. The average value of three independent experiments is shown. All data are expressed as the mean ± SD of the experiment. *p < 0.05, **p < 0.01, and ***p < 0.001 compared to the control group. (**C**) Intracellular lipids were stained with oil Red O (200× magnification) and quantified. Treatment with UDE and (+)-catechin resulted in reduced formation of adipocytes. The data are presented as the mean percentage compared to DMSO-treated cells. All data are expressed as the mean ± SD of the experiment. **p < 0.01 and ***p < 0.001 compared to the MDI control group.

Prior to investigating the anti-adipogenic effects of UDE and (+)-catechin, An MTT assay was performed to investigate the dose dependent cytotoxic effect in 3T3-L1 cells. Cells were treated with different concentrations of UDE (0-1,000 μg/ml) and (+)-catechin (0-200 μM) for 48 h, and then an MTT analysis was performed. According to the results, UDE and (+)-catechin had no cytotoxic effects at 500 μg/ml and 50 μM, respectively (Figs. 2A and 2B). In 3T3-L1 cells, the inhibitory effect on adipocyte differentiation was measured at non-toxic concentrations of UDE and (+)-catechin. To investigate the inhibitory effect of UDE on the differentiation of 3T3-L1 pre-adipocytes into adipocytes, pre-adipocytes were induced by treatment with MDI in cells containing or without UDE (10, 50, 75, 100, or 200 μg/ml) and (+)-catechin (50 μM) and quantified intracellular lipid accumulation over 8 days. As shown in Fig. 2B, adipocyte differentiation is associated with an increase in Oil Red O positive cells, whereas treatment with with UDE and (+)-catechin markedly inhibited lipid accumulation in differentiated adipocytes.

### Adipogenic Transcription Factor Production and Effect on Adipocyte Differentiation by UDE and (+)-Catechin

**Fig. 3:**
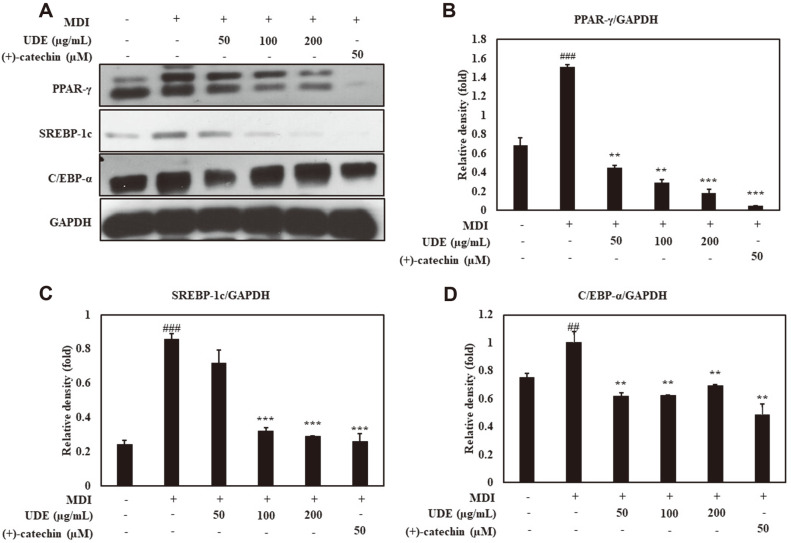
Effect of UDE and (+)-catechin on adipogenic transcription factors in 3T3-L1 cells. (**A**) 3T3-L1 cells were treated with UDE (50, 100, 200 μg/ml) or (+)-catechin (50 μM) for 48 h in adipocyte-induction media. Western blot using GAPDH as a loading control was performed for analysis of whole cell lysates. (**B**) Quantification of the band intensity ratios of PPAR-γ, (**C**) SREBP-1c, and (**D**) C/EBPα relative to GAPDH. The data are presented as the mean percentage compared to DMSO-treated cells. All data are expressed as the mean ± SD of the experiment. ^##^*p* < 0.01 and ^###^*p* < 0.001 compared to the control group; **p < 0.01 and ***p < 0.001 compared to the MDI control group.

**Fig. 4:**
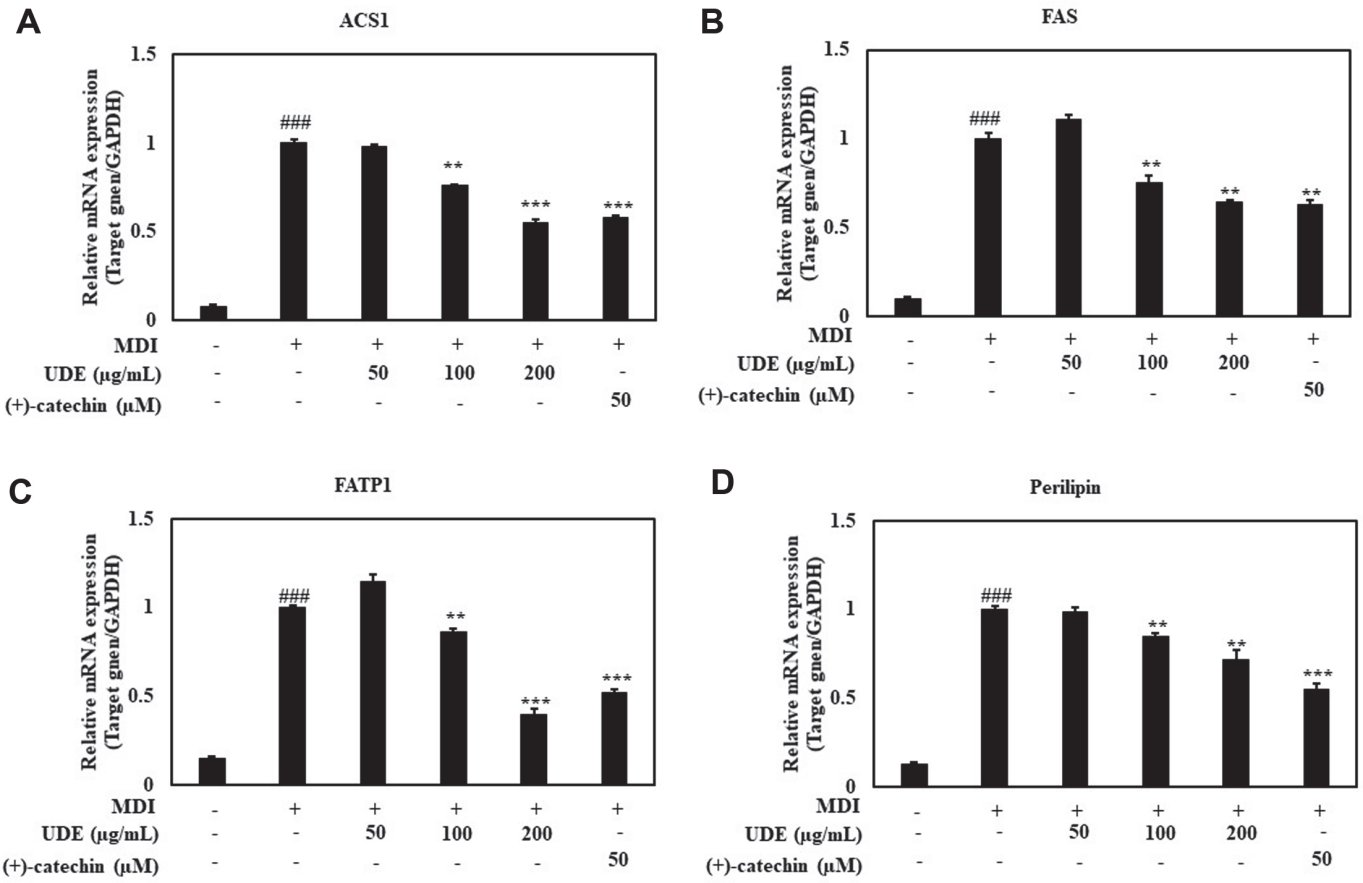
Effect of UDE and (+)-catechin on mRNA levels of proteins regulating synthesis, transport and storage of fatty acid. 3T3-L1 cells were treated with UDE (50, 100, and 200 μg/ml) or (+)-catechin (50 μM) for 48 h in adipocyte-induction media. Adipocyte RNA was isolated, and real-time PCR analysis was performed to determine mRNA expressions of ACS1, FAS, FATP1, and perilipin. GAPDH was used as an internal control. All data are expressed as the mean ± SD of the experiment. ^###^*p* < 0.001 compared to the control group; **p < 0.01 and ***p < 0.001 compared to the MDI control group.

To determine the effect of UDE and (+)-catechin on the adipogenic pathways, we evaluated the production of transcription factors PPARγ, C/EBPα, and SREBP-1c, as well as the protein levels of adipogenic transcription factors in 3T3-L1 cells. Substantially increased levels of PPARγ, C/EBPα, and SREBP-1c were observed in differentiated adipocytes compared with preadipocytes (Fig. 3). Significantly reduced PPARγ protein expression was observed at 50, 100, and 200 μg/ml of UDE and 50 μM (+)-catechin compared to control adipocytes. In addition, After treatment with 50, 100, and 200 μg/ml of UDE and 50 μM (+)-catechin, a reduced protein level of C/EBPα was observed. Treatment with UDE and (+)-catechin also resulted in decreased levels of SREBP-1c protein. In addition, tests for mRNA expression of ACS1, FAS and FATP1 were performed to determine the efficacy of UDE and (+)-catechin. Perilipin is a downstream target of the adipogenic transcription factor. As shown in Fig. 4, it was observed that the mRNA levels of these target genes increase during adipocyte differentiation. However, treatment with UDE (100 and 200 μg/ml) and (+)-catechin (50 μM) significantly reduced the mRNA expression of these genes.

### Effects of UDE and (+)-Catechin on HFD-Induced Obese Mice on Body Weight Gain, Adipose Tissue Weight, and Adipocyte Size

**Fig. 5:**
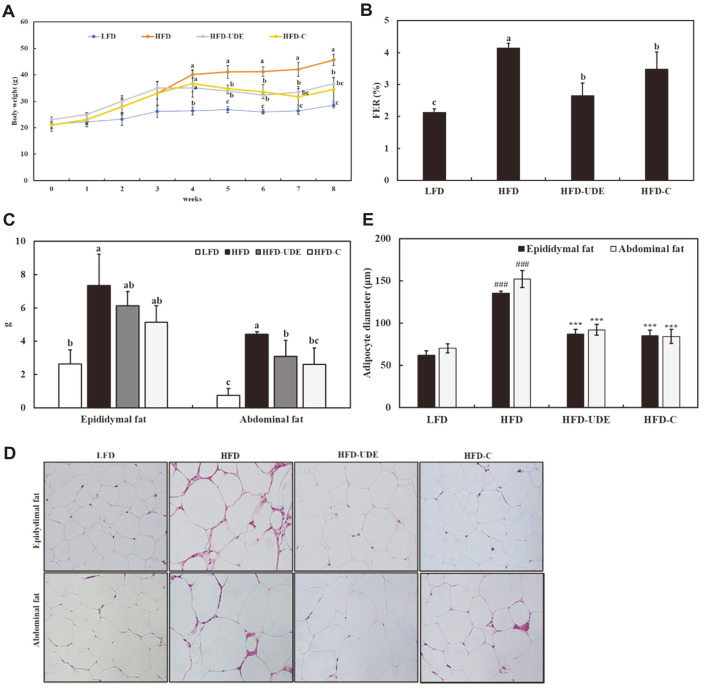
Regulation of body weight gain, food efficiency ratio (FER), weight and histology of adipose tissue by UDE in high-fat diet (HFD)-fed mice. (**A**) Body weight gain at the end of the treatment period. (**B**) FER as the body weight gain (g/day) divided by food intake (g/day). (**C**) White adipose tissue weights in HFD-fed mice. All values are expressed as mean ± SD (*n* = 6). Different letters are significantly different at *p* < 0.05 by Duncan’s multiple range test. (**D**) Adipose tissue histology. Representative adipose tissue sections stained with hematoxylin-eosin (original magnification ×200). (**E**) Average diameter of adipocytes in adipose tissue from each group. All data are expressed as the mean ± SD of the experiment. ^###^*p* < 0.001 compared to the LFD group; ***p < 0.001 compared to the HFD control group.

To examine the in vivo effects of UDE, 200 mg/kg per day UDE and 30 mg/kg per day (+)-catechin were orally administered once daily for 4 weeks to C57BL/6J mice. Prior to the initiation of treatment, significantly (*p* < 0.05) increased body weight was observed in all HFD groups compared with the low-fat diet (LFD) group (Fig. 5A). However, lower body weights were observed in the HFD-UDE and HFD-C groups compared to the HFD group (1.57 ± 1.21 g and -2.24 ± 3.95 g vs. 5.46 ± 0.92 g, respectively; *p* < 0.05). The FER is shown in Fig. 5B. A significantly higher FER was observed in the HFD group compared to the LFD group, but decreased in the HFD-UDE and HFD-C groups compared to the HFD group. As shown in Fig. 5C, reduced epididymal and abdominal adipose tissue weight was observed in the HFD-UDE and HFD-C groups compared to the HFD group. In particular, the weight of abdominal adipose tissue decreased by 30% and 41% after UDE and (+)-catechin treatment., respectively (*p* < 0.05). Histological analysis showed that UDE and (+)-catechin treatment also reduced mean epididymal and abdominal fat cell sizes in the HFD group. (Fig. 4D). A significantly higher FER (%) was observed in the HFD group compared to the LFD group, and body weight, fat weight and fat cell size increased. Therefore, even if the same amounts of an LFD and HFD are consumed, body weight and fat weight are increased with an HFD. However, UDE has the effect of reducing fat weight and adipocyte size by reducing FER.

### Effects of UDE and (+)-Catechin on HFD-Induced Obese Mice on Serum ALT, AST, and Lipid Levels

**Fig. 6:**
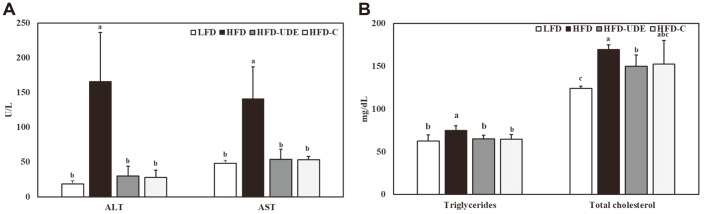
Changes in circulating AST, ALT, triglycerides, and total cholesterol by UDE in high-fat diet-fed mice. (**A**) Serum concentrations of AST and ALT, and (**B**) triglycerides and total cholesterol measured as mean ± SD (*n* = 6). Different letters are significantly different at *p* < 0.05 by Duncan’s multiple range test.

Serum ALT and AST levels were measured to assess liver toxicity of UDE and (+)-catechin, which were significantly higher in the HFD group compared to the LFD group (Fig. 6A). Compared to the HFD group, the group treated with UDE or (+)-catechin had significantly lower serum levels of ALT and AST, which means that UDE and (+)-catechin may not be toxic to experimental animals at the concentrations used in this study. Serum levels of TGs and TCs in experimental animals increased by 19.8% and 36.9%, respectively, in the HFD group compared to the serum levels in the LFD group (Fig. 6B). However, TG and TC levels were significantly reduced in the group administered UDE and (+)-catechin. Serum TG and TC levels decreased by 13.6% and 11.5% in the HFD-UDE group, respectively, compared to that in the HFD group.

### Effects of UDE and (+)-Catechin on Hepatic Steatosis in HFD-Induced Obese Mice

**Fig. 7:**
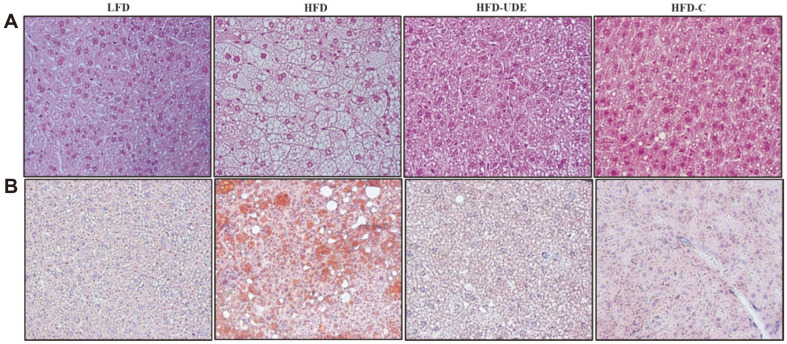
Inhibition of hepatic lipid accumulation by UDE in high-fat diet-fed mice. (**A**) Representative liver sections stained with hematoxylin-eosin (original magnification × 200). (**B**) Representative liver sections stained with Oil Red O (original magnification × 200).

H&E and oil red O staining of liver tissue are shown in Fig. 7. Liver sections from the LFD group presented a normal hepatic cell morphology. In contrast, white lipid droplet deposition in hepatocytes was observed in tissues from the HFD. However, a smaller amount of hepatic fat accumulation was observed in the group treated with UDE or (+)-catechin compared to the HFD group (Fig. 7A). Oil red O staining showed more red spots and lipid droplets than in the HFD group compared to the LFD group. However, red spots and lipid droplets were reduced in the UDE or (+)-catechin-treated group compared to the HFD group (Fig. 7B).

### Protein Expression Related to Lipid Metabolism in Liver of HFD-induced Obese Mice

**Fig. 8:**
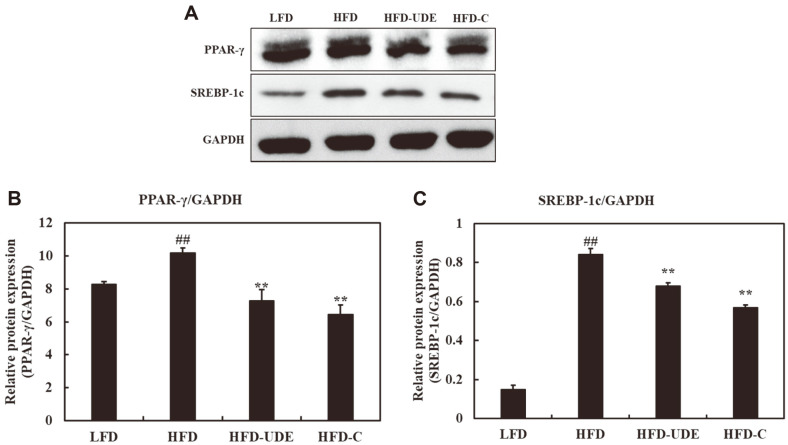
Effect of UDE and (+)-catechin on adipogenic transcription factors in liver tissue. (**A**) Western blot analysis using GAPDH as the loading control was performed for measurement of PPAR-γ and SREBP-1c protein expression in liver tissue. (**B**) Quantification of the band intensity ratios of PPAR-γ and (**C**) SREBP-1c relative to GAPDH. The data are presented as the mean percentage compared to DMSO-treated cells. All data are expressed as the mean ± SD of the experiment. ##*p* < 0.01 compared to the LFD group; **p < 0.01 compared to the HFD group.

In this study, we analyzed the expression of SREBP-1c and PPAR-γ in liver tissue isolated from the LFD, HFD, HFD-UDE and HFD-C groups to determine if the reduced fat amount is related to the SREBP-1c and PPAR-γ signaling pathways. Significantly reduced expression of SREBP-1c and PPAR-γ was observed in the HFD-UDE and HFD-C groups compared to the HFD group. (Fig. 8).

## Discussion

*U. davidiana* var. *japonica* has been reported to contain important phytochemicals such as flavonoids [15], triterpene esters [16], sesquiterpene-*O*-naphthaquinones [17], lignan and neolignan glycosides [18], and butenyl cyclohexenone glycosides [19], which exert pharmacological activities, including anti-oxidative effects [17], Inhibitory effect on collagen-induced arthritis [20], modulatory effect on immunity [21], anti-angiogenic activity [22]. Several medicinal therapeutic effects of *U. davidiana* var. *japonica* have been demonstrated in many studies, however the anti-obesity and lipid metabolism effects have not been evaluated. The main objective of this study was to investigate the anti-obesity and lipid metabolism effects of UDE and to elucidate the mechanism using 3T3-L1 preadipocytes and an HFD-induced obese murine model.

First, we identified one active component ((+)-catechin) from the UDE and performed HPLC to quantify its contents. Antioxidant and anti-inflammatory effects of (+)-catechin have been reported as well as its involvement in the regulation of blood sugar levels and other properties [23-26]. In particular, Yang *et al*. [27] C/EBP/PPARγ/SREBP-1c and cAMP/PKA signaling pathways were regulated to confirm the anti-obesity and regulatory effects of (+)-catechin on lipid metabolism by inhibiting adipocyte differentiation. These results suggest that (+)-catechin can effectively suppress obesity by regulating lipid metabolism.

Excessive weight gain is closely related to an increase in body fat mass, which can be caused by an increase in the size (hypertrophy) and number of fat cells (hypertrophy). Therefore, inhibition of adipogenesis is an important target for the prevention and treatment of obesity [28, 29]. Adipogenic differentiation of 3T3-L1 pre-adipocytes can mimic adipocyte proliferation in vivo, so these cells are the most used in vitro studies for adipogenesis and fat metabolism [30].

Differentiated 3T3-L1 adipocytes have morphological and biochemical properties similar to adipocytes in vivo, and also partially respond to adipogenic hormones and metabolic regulators [31]. In addition, the advantages of 3T3-L1 cells include simplicity and infinite availability, showing a fairly good correlation in connection with animal studies. For example, inhibition of 3T3-L1 differentiation has been correlated with weight and body fat mass reduction in an in vivo model [32, 33]. Therefore, in this study, the anti-adipogenic effect and mechanism of UDE and (+)-catechin were investigated using 3T3-L1 cells, and the anti-obesity effect was further investigated in C57BL/6J mice fed with HFD. We investigated the effect of UDE and (+)-catechin on adipocyte differentiation in Curly venom. Significant lipid droplets were observed in the 3T3-L1 control cells after adipocyte differentiation. However, UDE and (+)-catechin treatment reduced lipid accumulation in 3T3-L1 adipocytes (Fig. 2B).

Adipogenesis relies on cell-to cell communication between cells and their surrounding environment [34]. The molecular events of adipogenesis are still unknown, but various factors involved in this cellular process have been recognized. Some adipogenesis stimulants include PPARγ, C/EBPα, C/EBPβ, C/EBPδ, and SREBP-1c [14]. These stimulants play an important role in stimulating the expression of various genes involved in adipocyte differentiation and maturation, including ACS1, FAS, FATP1, and perilipin [35, 36]. PPARγ and C/EBPα are characterized by regulating cell growth inhibition of adipocyte proliferation, and act as master regulators that induce differentiation by regulating several adipocyte-specific genes also [35]. SREBP-1c regulates lipid homeostasis by regulating several genes involved in fatty acid synthesis, and overexpression of this gene provides the production of PPARγ ligands [36]. In this study, we studied the effect on the expression of major transcription factors by UDE and (+)-catechin in the adipogenesis pathway. We found that UDE and (+)-catechin inhibited protein expression of the adipogenic transcription factors PPARγ, C/EBPα, and SREBP-1c in 3T3-L1 pre-adipocytes. It has been shown to inhibit adipocyte differentiation by reducing the formation of lipid droplets (Fig. 3). In addition, the lipid genes ACS1, FAS, FATP1, and lipid droplet formation-related protein perilipin significantly increased the mRNA expression of each gene upon induction of differentiation, but significantly decreased when treated with UDE and (+)-catechin (Fig. 4). UDE and (+)-catechin produce lipid droplets by inhibiting the expression of adipogenic transcription factors PPARγ, SREBP-1c, and C/EBPα, and genes involved in lipid synthesis, lipid transport, and lipid storage in 3T3-L1 adipocytes. It is thought that the differentiation into adipocytes can be suppressed by reducing.

We also demonstrated the remarkable effect of oral administration of UDE in HFD-induced obese mice. UDE and (+)-catechin treatment significantly reduced weight gain and FER values in HFD-induced obese mice, suggesting that mice treated with UDE and (+)-catechin are less efficient at converting food into energy than the untreated HFD mice. Therefore, weight loss in the UDE and (+)-catechin treatment groups may not have occurred due to decreased food intake. Obesity is the result of hypertrophy and hyperplasia. In this study, smaller epididymal and abdominal adipocytes were observed after UDE treatment. Thus, UDE may effectively inhibit epididymal and abdominal adipocyte hypertrophy in the HFD-induced obese mouse. In adipocyte hypertrophy, obesity indicates a dysfunction of adipose tissue and is highly associated with many metabolic syndromes, including nonalcoholic fatty liver disease, atherosclerosis, dyslipidemia, type 2 diabetes, and insulin resistance [37-39]. It suggests that UDE may reduce metabolic disease by inhibiting adipocyte hypertrophy.

Animals given HFD not only increase body fat mass, but also adversely affect plasma and liver lipids [40]. In addition, visceral fat can be delivered to the liver, and within hepatocytes it releases free fatty acids that can be maintained as TG, so reducing the amount of visceral fat is closely related to promoting liver steatosis [41]. In general, the increase in liver size and weight in vivo increases with HFD intake through accumulation of cholesterol and triglycerides [41, 42]. In particular, the reduction in body fat mass is closely related to the improvement of fatty liver. (liver steatosis). Histologically, abnormal hepatic steatosis and larger lipid droplets were observed in the HFD group; however, less hepatic fat accumulation was observed in the HFD-UDE group compared to the HFD group (Fig. 7). The results of our study suggest that UDE improves circulating lipid profile and hepatic steatosis, at least in part, in HFD-induced obese mice through reduction in fat pad (epididymal and abdominal) mass.

Excessive expression of SREBP-1c significantly increases liver TG and TC, leading to the development of hepatic steatosis [44]. Therefore, inhibition of hepatic SREBP-1c expression may attenuate hepatic steatosis. In the current study, markedly elevated hepatic SREBP-1c protein expression was observed in HFD-induced obese mice, however consumption of UDE resulted in suppressed expression, resulting in a low rate of hepatic lipid synthesis and TG and TC content. These results suggest that dietary UDE decreased the hepatic lipid accumulation and TG and TC levels through regulation of the SREBP-1c-mediated gene transcription pathway. We also elucidated the UDE-mediated anti-obesity molecular mechanisms. PPARγ is a transcription factor, an important transcription factor for adipocyte differentiation that stimulates the expression of genes required for adipogenesis [14]. Reduced expression of PPARγ resulted in inhibition of 3T3-L1 differentiation, which may have prevented weight gain in HFD-induced obese mice. Decreased expression of PPARγ and target genes were also observed in liver tissues of UDE-treated mice. Thus, the anti-obesity effect of UDE may be due in part to the downregulation of PPARγ.

In conclusion, UDE treatment at least partially inhibited adipocyte differentiation of 3T3-L1 preadipocytes through down-regulation of adipogenic transcription factors PPARγ, C/EBPα, and SREBP-1c. In addition, UDE treatment reduced body fat mass and improved plasma lipid profile in HFD-induced obese mice. Thus, UDE is a viable option for the treatment of several related metabolic disorders, including obesity and hyperlipidemia.

## Figures and Tables

**Table 1:** Sequences of the primers used for real-time PCR.

Target	Primer sequences
GAPDH	5’-GTATGATCCACTCACGGCA-3’
	5’-GGTCTCGCTCCTGGAAGAGG-3’
ACS1	5’-GTCTTTGCCACATCCGACCTATC-3’
	5’-TTAGTGCAAACCCAGTTGTGCTTC-3’
FAS	5’-AGCACTGCCTTCGGTTCAGTC-3’
	5’-AAGAGCTGTGGAGGCCACTTG-3’
FATP1	5’-CAGACGGACGTGGCTGTGTA-3’
	5’-GCCGAGCATAGGATGCAAGAA-3’
Perilipin	5’-GATGAGAGCCATGACGACCAGA-3’
	5’-TGTGTACCACACCACCCAGGA-3’

Acyl-CoA synthetase-1 (ACS1), fatty acid synthesis (FAS), fatty acid transport-1 (FATP1)

## References

[1] Park JP, Kim JH, Park MK, Yun JW (2011). Potential agents for cancer and obesity treatment with herbal medicines from the green garden. Biotechnol. Bioprocess.

[2] Lee SJ, Oh PS, Ko JH, Lim K, Lim KT (2006). Protective effect of glycoprotein isolated from *Ulmus davidiana* Nakai on carbon tetrachloride-induced mouse liver injury. J. Pharm. Pharmacol..

[3] Jun CD, Pae HO, Kim YC, Jeong SJ, Yoo JC, Lee EJ (1998). Inhibition of nitric oxide synthesis by butanol fraction of the methanol extract of *Ulmus davidiana* in murine macrophages. J. Ethnopharmacol..

[4] Jin UH, Suh SJ, Kim KS, Kim JK, Kim MS, Kwon DY (2007). Antiinflammatory effects of *Ulmus davidiana* Planch (Ulmaceae) on collagen-induced arthritis in rats. Environ. Toxicol. Pharmacol..

[5] Kang SK, Kim KS, Byun YS, Suh SJ, Jin UH, Kim KH (2006). Effects of *Ulmus davidiana* Planch on mineralization, bone morphogenetic protein-2, alkaline phosphatase type I collagen, and collagenase-1 in bone cells. In Vitro Cell Dev. Biol. Anim..

[6] Suh SJ, Yun WS, Kim KS, Jin UH, Kim JK, Kim MS (2007). Stimulative effect of *Ulmus davidiana* Planch (Ulmaceae) on osteoblastic MC3T3-E1 cells. J. Ethnopharmacol..

[7] Lee GY, Jang DS, Kim J, Kim CS, Kim YS, Kim JH (2008). Falvan-3-ols from *Ulmus davidiana* var. japonica with inhibitory activity on protein glycation. Planta Med..

[8] Shin DY, Kim HS, Min KH, Hyun SS, Kim SA, Huh H (2000). Isolation of a potent anti-MRSA sesquiterpenoid quinine from *Ulmus davidiana* var. japonica. Chem. Pharm. Bull. (Tokyo).

[9] Hamilton MT, Hamilton DG, Zderic TW (2007). Role of low energy expenditure and sitting in obesity, metabolic syndrome, type 2 diabetes, and cardiovascular disease. Diabetes.

[10] Lee MS, Kim IH, Kim CT, Kim Y (2011). Reduction of body weight by dietary garlic is associated with an increase in uncoupling protein mRNA expression and activation of AMP-activated protein kinase in diet-induced obese mice. J. Nutr..

[11] Yun JW (2010). Possible anti-obesity therapeutics from nature - a review. Phytochemistry.

[12] Birari R, Javia V, Bhutani KK (2010). Antiobesity and lipid lowering effects of *Murraya koenigii* (L.) Spreng leaves extracts and mahanimbine on high fat diet induced obese rats. Fitoterapia.

[13] Kang CH, Kwon YJ, So JS (2014). Anti-adipogenic effects of Corni fructus in 3T3-L1 preadipocytes. Biotechnol. Bioprocess..

[14] Nerurkar PV, Lee Y, Nerurkar VR (2010). *Momordica charantia* (bitter melon) inhibits primary human adipocyte differentiation by modulating adipogenic genes. BMC Complement. Altern. Med..

[15] Son BW, Park JH, Zee OP (1989). Catechin glycoside from *Ulmus davidiana*. Arch. Pharm. Res..

[16] Lee MK, Kim YC (2001). Five novel neuroprotective triterpene esters of *Ulmus davidiana* var. *japonica*. J. Nat. Prod..

[17] Kim JP, Kim WG, Koshino H, Jung J, Yoo ID (1996). Sesquiterpene-*O*-naphthaquinones from the root bark of *Ulmus davidiana*. Phytochemistry.

[18] Lee MK, Sung SH, Lee HS, Cho JH, Kim YC (2001). Lignan and neolignan glycosides from *Ulmus davidiana* var. *japonica*. Arch. Pharm. Res..

[19] Zheng MS, Lee YK, Li Y, Hwangbo K, Lee CS, Kim JR (2010). Inhibition of DNA topoisomerases I and II and cytotoxicity of compounds from *Ulmus davidiana* var. *japonica*. Arch. Pharm. Res..

[20] Kim KS, Lee SD, Kim KH, Kil SY, Chung KH, Kim CH (2005). Suppressive effects of a water extract of *Ulmus davidiana* Planch (Ulmaceae) on collagen-induced arthritis in mice. J. Ethnopharmacol..

[21] Lee Y, Park H, Ryu HS, Chun M, Kang S, Kim HS (2007). Effects of elm bark (*Ulmus davidiana* var. *japonica*) extracts on the modulation of immunocompetence in mice. J. Med. Food..

[22] Jung HJ, Jeon HJ, Lim EJ, Ahn EK, Song YS, Lee S (2007). Anti-angiogenic activity of the methanol extract and its fraction of *Ulmus davidiana* var. *japonica*. J. Ethnopharmacol..

[23] Bhardwaj P, Khanna D (2013). Green tea catechins: defensive role in cardiovascular disorders. Chin. J. Nat. Med..

[24] Gruz J, Ayaz FA, Torun H, Strnad M (2011). Phenolic acid content and radical scavenging activity of extracts from medlar (*Mespilus germanica* L.) fruit at different stages of ripening. Food Chem..

[25] Terra X, Pallarés V, Ardèvol A, Bladé C, Fernández-Larrea J, Pujadas G (2011). Modulatory effect of grape-seed procyanidins on local and systemic inflammation in diet-induced obesity rats. J. Nutr. Biochem..

[26] Tian Y, Zou B, Yang L, Xu SF, Yang J, Yao P (2011). High molecular weight persimmon tannin ameliorates cognition deficits and attenuates oxidative damage in senescent mice induced by D-galactose. Food Chem. Toxicol..

[27] Yang J, Shijie D, Feng L, Chen Z, Dongxiao SW, Yilun C (2019). Effects of (+)-catechin on the differentiation and lipid metabolism of 3T3-L1 adipocytes. J. Funct. Foods.

[28] Otto TC, Lane MD (2005). Adipose development: from stem cell to adipocyte. Crit. Rev. Biochem. Mol. Biol..

[29] Tang QQ, Otto TC, Lane MD (2003). Mitotic clonal expansion: a synchronous process required for adipogenesis. Proc. Natl. Acad. Sci. USA.

[30] Tang QQ, Otto TC, Lane MD (2003). CCAAT/enhancer-binding protein β is required for mitotic clonal expansion during adipogenesis. Proc. Natl. Acad. Sci. USA.

[31] MacDougald OA, Lane MD (1995). Adipocyte differentiation: when precursors are also regulators. Curr. Biol..

[32] Li ZS, Noda K, Fujita E, Manabe Y, Hirata T, Sugawara T (2015). The green algal carotenoid siphonaxanthin inhibits adipogenesis in 3T3-L1 preadipocytes and the accumulation of lipids in white adipose tissue of KK-Ay mice. J. Nutr..

[33] Min B, Lee H, Song JH, Han MJ, Chung J (2014). Arctiin inhibits adipogenesis in 3T3-L1 cells and decreases adiposity and body weight in mice fed a high-fat diet. Nutr. Res. Pract..

[34] Cornelius P, MacDougald OA, Lane MD (1994). Regulation of adipocyte development. Annu. Rev. Nutr..

[35] Rosen ED, Walkey CJ, Puigserver P, Spiegelman BM (2000). Transcriptional regulation of adipogenesis. Genes Dev..

[36] Lefterova MI, Lazar MA (2009). New developments in adipogenesis. Trends Endocrinol. Metab..

[37] Kim J, Lee H, Lim J, Oh J, Shin SS, Yoon M (2017). The angiogenesis inhibitor ALS-L1023 from lemon-balm leaves attenuates high-fat diet-induced nonalcoholic fatty liver disease through regulating the visceral adipose-tissue function. Int. J. Mol. Sci..

[38] Oh J, Lee H, Lim H, Woo S, Shin SS, Yoon M (2015). The herbal composition GGEx18 from *Laminaria japonica*, *Rheum palmatum*, and *Ephedra sinica* inhibits visceral obesity and insulin resistance by upregulating visceral adipose genes involved in fatty acid oxidation. Pharm. Biol..

[39] Laforest S, Labrecque J, Michaud A, Cianflone K, Tchernof A (2015). Adipocyte size as a determinant of metabolic disease and adipose tissue dysfunction. Crit. Rev. Clin. Lab. Sci..

[40] Surwit RS, Feinglos MN, Rodin J, Sutherland A, Petro AE, Opara EC (1995). Differential effects of fat and sucrose on the development of obesity and diabetes in C57BL/6J and A/J mice. Metabolism.

[41] Kabir M, Catalano KJ, Ananthnarayan S, Kim SP, Van Citters GW, Dea MK (2005). Molecular evidence supporting the portal theory: a causative link between visceral adiposity and hepatic insulin resistance. Am. J. Physiol. Endocrinol. Metab..

[42] Klipsic D, Landrock D, Martin GG, McIntosh AL, Landrock KK, Mackie JT (2015). Impact of SCP-2/SCP-x gene ablation and dietary cholesterol on hepatic lipid accumulation. Am. J. Physiol. Gastrointest. Liver Physiol..

[43] Banerji MA, Buckley MC, Chaiken RL, Gordon D, Lebovitz HE, Kral JG (1995). Liver fat, serum triglycerides and visceral adipose tissue in insulin-sensitive and insulin-resistant black men with NIDDM. Int. J. Obes. Relat. Metab. Disord..

[44] Ahmed MH, Byrne CD (2007). Modulation of sterol regulatory element binding proteins (SREBPs) as potential treatments for non-alcoholic fatty liver disease (NAFLD). Drug Discov. Today.

